# Acute Myocardial Infarct in a Young Bodybuilder Taking Anabolic Steroids: A Case Report

**DOI:** 10.7759/cureus.95790

**Published:** 2025-10-31

**Authors:** Nisrine N Makarem, Mohammad El Jammal

**Affiliations:** 1 Family Medicine, American University of Beirut Medical Center, Beirut, LBN

**Keywords:** acute myocardial infarction in young patients, anabolic-androgenic steroids, cad: coronary artery disease, non-stemi, performance-enhancing drugs

## Abstract

We describe a case report of a 35-year-old bodybuilder suffering from an acute myocardial infarct (AMI). He had been taking trenbolone acetate, testosterone enanthate, and nandrolone for six weeks. The coronary angiogram showed an 80% lesion in the mid-segment of the obtuse marginal artery. A few cases of AMI in athletes taking anabolic steroids (AS) have been reported. Physicians should always consider and inquire about AS use as a potential culprit among athletes with AMI. Multiple plausible mechanisms explain the pathophysiology of AMI in AS users.

## Introduction

Anabolic steroids (AS), such as testosterone, belong to a class of performance-enhancing drugs. Unfortunately, AS misuse is extremely common globally, with an estimated lifetime prevalence ranging between 1-5% [1]. In Lebanon, a study reported a prevalence rate for AS use at around 10.7% in adult fitness center attendees who are not professional or career athletes [2]. Until the early 1980s, AS were primarily used by professional athletes. However, a shift occurred as recreational athletes began using AS to enhance muscle strength, improve physical appearance, and boost performance [3,4]. Despite publicity given to abuse of AS among elite athletes, the highest level of abuse is among recreational athletes [5]. This practice is usually characterized by 6-12-week cycles of administration, including the stacking of different AS compounds at doses 50-1000 times higher than those recommended for therapeutic purposes. Most of the toxicity associated with AS use is related to this high dosage. Rather than consulting clinicians, users typically seek information about these drugs from other athletes, physical trainers at the gym, or the internet.

AS abuse has been associated with serious side effects, including cardiovascular adverse events, hepatotoxicity, hypogonadism, and psychiatric disorders [6]. Anabolic steroids exert adverse cardiac effects through mechanisms such as lipid imbalance, endothelial dysfunction, and a heightened risk of thrombosis. Concerning the cardiovascular effects of AS abuse, acute myocardial infarction (AMI) is possibly the most devastating one. AS use is rarely linked to AMI, and occurrences of AMI secondary to AS misuse have been limited to case reports. Most cases of AMI occur in patients with a background of hyperlipidemia, hypertension, and coronary artery disease (CAD).

Here, we present the case of a young previously healthy man who developed a non-ST-elevation myocardial infarction (NSTEMI) following anabolic steroid use, in the absence of atheromatous or thrombotic disease. In this case report, we describe AS misuse as the most likely cause of AMI requiring intervention at a young age. We will detail pertinent findings of the patient’s history, physical examination, and further investigations that may explain our patient’s findings.

## Case presentation

A 35-year-old male with no prior medical history presented to the clinic as a drop-in with an acute substernal burning sensation that began two hours earlier. The pain occurred at rest, was localized, non-radiating, and was not preceded by any known cause. Notably, the patient’s father and grandfather both had CAD in their 50s. Moreover, he had a 30-pack-year smoking history. He reported using AS for the past six weeks. He reported taking twice-weekly intramuscular injections of nandrolone 200 mg/ml, trenbolone acetate 100mg/ml, and testosterone enanthate 250 mg/ml, a regimen prescribed by his gym personal trainer.

**Figure 1:**
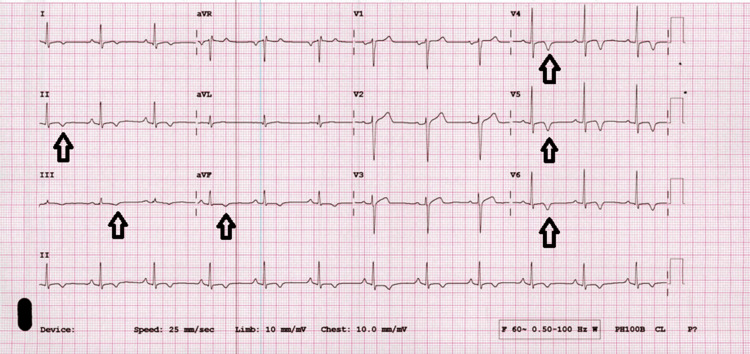
A 12-lead electrocardiogram showing T-wave inversion in leads V4-V5-V6, II, III, and aVF Arrows indicate T-wave invesrion in leads V4-V5-V6, II, III, and aVF

The patient was referred to the emergency department. He had a blood pressure of 145/83 mmHg, a heart rate of 71 beats/min, a respiratory rate of 15/min, and a temperature of 36°C. Arterial oxygen saturation was 99%. On physical exam, his lungs were clear, he had palpable pulses, and no jugular venous distension or lower extremity edema. A 12-lead electrocardiogram showed T-wave inversion in leads V4-V6, I, II, and aVF (Figure 1). The patient was started on oral therapy with aspirin (300mg once), clopidogrel (600 mg once), esomeprazole (40 mg), and rosuvastatin (10 mg), and subcutaneous therapy with heparin.

His blood chemistry revealed an elevated cardiac troponin-I level of 0.043 ng/ml (0.000 - 0.030 ng/ml). His complete blood count, baseline kidney, and liver function tests were within normal limits. A fasting lipid profile revealed high-density lipoprotein-cholesterol (HDL-C) of 33 mg/dl (normal: > 45 mg/dl, borderline: 36-45 mg/dl, high risk: < 35 mg/dl) and low-density lipoprotein-cholesterol (LDL-C) of 114 mg/dl (very high risk: < 70 mg/dl, high risk: <100 mg/dl, moderate and low risk: < 130 mg/dl). A coagulative workup, including antithrombin III, factor V Leiden deficiency, protein C and S activity, and an autoimmune workup, was not done.

**Figure 2:**
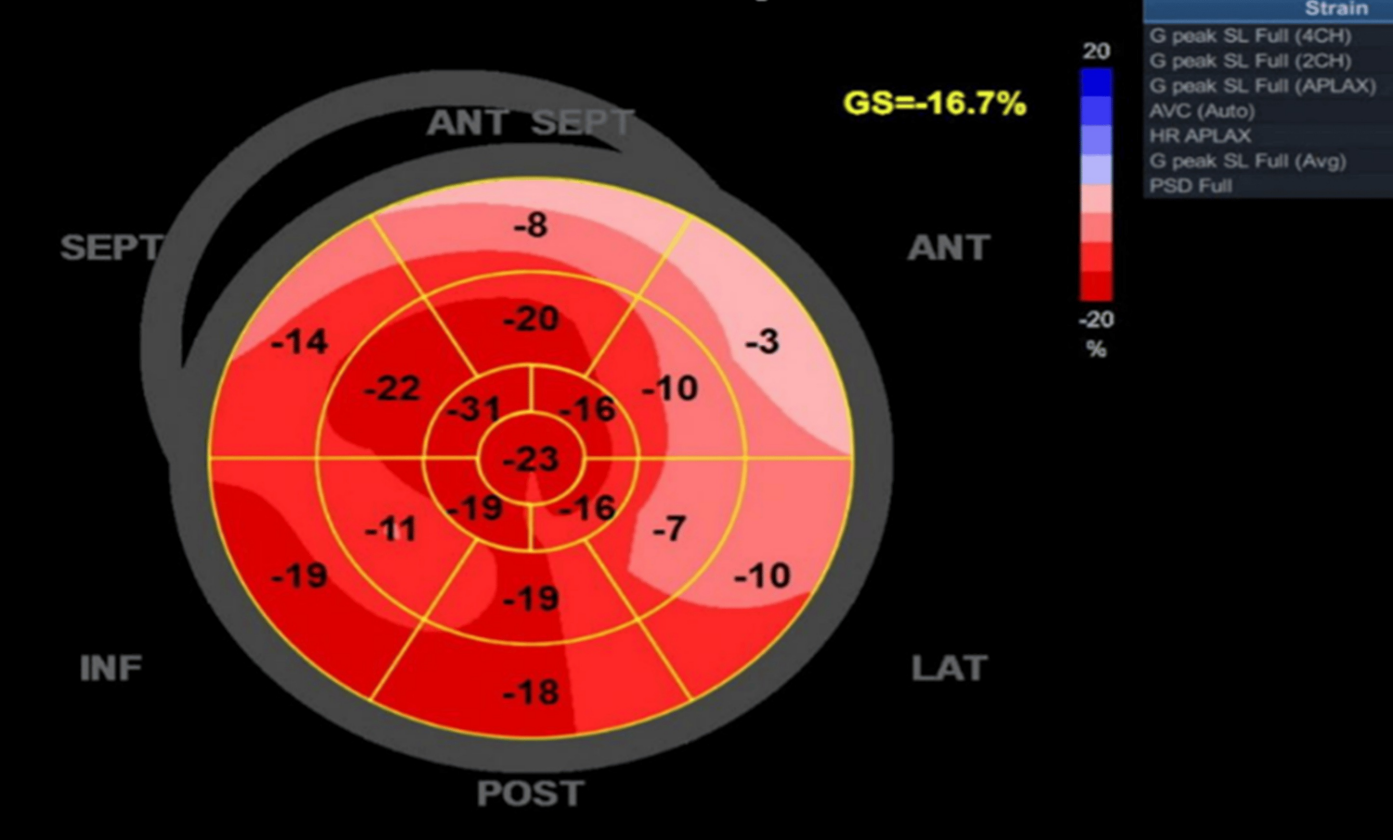
The GLS pattern shows reduced strain in the lateral and anterior segments, consistent with the circumflex artery territory. Dark red (deep red), indicating a normal longitudinal strain and healthy myocardial deformation (normal contraction). Light red to pink (−10% to −5%), indicating reduced strain / hypokinetic myocardium (reduced contractility). Pale pink (near white) (0% to −3%), indicating severely reduced or absent strain and akinetic or dyskinetic myocardium (no proper contraction).

In the emergency department, a transthoracic echocardiography was performed and showed normal left ventricular systolic function, with an estimated left ventricular ejection fraction of 60-64%. The GLS pattern obtained by echocardiography showed a reduced strain in the lateral and anterior segments, consistent with the circumflex artery territory (Figure 2).

**Figure 3:**
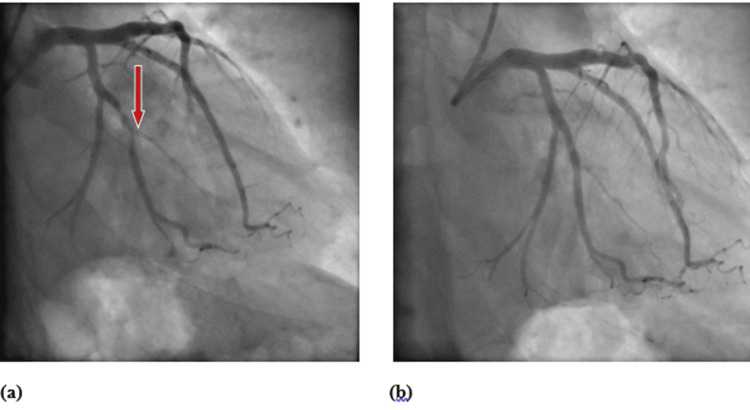
Coronary angiogram before (a) and after (b) placing the stent in the first obtuse marginal branching off the circumflex coronary artery.

At that point, a decision was made to proceed with a coronary angiogram. The left main coronary artery was unremarkable. However, the left anterior descending coronary artery was mildly, diffusely diseased with luminal irregularities in the proximal, mid, and distal segments. The circumflex coronary artery had a 30% stenosis in its mid-segment with mild luminal disease in its proximal and distal segments. It gave rise to a large first obtuse marginal branch with a 50% lesion in its proximal segment and an 80% lesion in its mid segment with mild luminal irregularities. As such, angioplasty was performed. A 3.0 × 28 mm drug-eluting stent was implanted across the lesions in the proximal and mid segments of the first obtuse marginal coronary artery; one inflation was performed with excellent angiographic results. Figure 3 shows the coronary angiogram before and after placing the stent.

During hospitalization, the patient remained electrically and hemodynamically stable, without pain recurrence and with analytical improvement. The patient was discharged after three days on dual antiplatelet therapy, which was to be continued for one year. Smoking cessation and risk factor optimization (stopping steroid use) were advised. He followed up at several clinics, and a thorough workup was done of his kidney function, thyroid function, as well as lipid profile. No major abnormality was found, aside from an elevated creatinine level (Cr 1.44), which was attributed to the contrast material given during the cardiac angiography. Troponin levels repeated two weeks later were normal. Aside from his positive family history and smoking status, AS abuse was considered to be a predisposing, albeit not the sole, cause of AMI in this young bodybuilder.

## Discussion

Methodology

A literature search based on PubMed listings up to 15 December 2023 using the search terms ‘anabolic steroids AND myocardial infarction’ identified 95 articles. Moreover, we examined the reference list of the articles identified by this search strategy and selected those we judged relevant.

Epidemiological findings on AS misuse

The estimated lifetime prevalence rates of AS usage in the general population vary, with higher rates found in the United States [7], certain regions in Europe and the Middle East, and lower rates in other parts of Asia and Africa [5]. Specific subgroups, such as former elite power athletes, recreational athletes, injecting drug users, and arrestees/prisoners, tend to report a higher lifetime prevalence of AS use compared to the general population [8]. The worldwide occurrence of AS utilization has been approximated to be around 3.3% to 6.4% among males and approximately 1.3% among females [9]. Although women have less global use of anabolic steroids, a study of North American high school students showed a 5.3% use rate [10].

Adverse effects of AS misuse

AS can have adverse effects on multiple organ systems, including endocrine, dermatological, skeletal, genitourinary, psychiatric, metabolic, hepatic, and cardiovascular systems [11, 12]. Most evidence on adverse side effects comes from case reports since it is unethical to give high doses of AS to induce side effects in research studies. Side effects range from mild, such as acne, to more serious and even potentially fatal complications, such as cardiovascular events, liver failure, and cancer [13]. A case series by Stefanou et al. reported pectoralis major rupture linked to AS use among bodybuilders [14].

Epidemiology of AS-related AMI

Studies have pointed to the link between using AS and a higher risk of MI [15]. Numerous case reports of AS-related AMI in otherwise healthy people to date included patients who were mostly young male athletes, and after ruling out all other potential causes of AMI, AS misuse was the only possible remaining explanation [4, 16-22]. Most of the case reports involve men aged 25-35 who are bodybuilders and have few or no cardiac risk factors for AMI.

A case report published by Melhem et al. documented a 26-year-old farmer and amateur bodybuilder who developed a Killip Class I AMI in the anterior wall while using AS. His lipid and hormone levels were elevated a few days before the event. After left anterior descending branch angioplasty, he had significant electrocardiographic sequelae and ventricular dysfunction. The patient was treated with primary angioplasty [4]. Another case study featured a 30-year-old bodybuilder who suffered an AMI while taking testosterone and stanozolol. Despite the absence of underlying atherosclerosis, coronary angiography revealed a substantial thrombotic burden in the left anterior descending artery [17]. A case report outlined the intricate medical history of a 25-year-old individual who, after six months of steroid use, was diagnosed with myocardial infarction, dyslipidemia, obesity, hyperuricemia, secondary diabetes, and chronic renal disease [23]. Additionally, another case report of a 26-year-old physically active physician with no coronary risk factors who presented with chest pain was initially misdiagnosed as skeletal muscle pain. He was later admitted to the hospital, where he suffered from a massive AMI. His history revealed chronic use of anabolic steroids and amino acid supplements, leading to increased cardiac markers and dynamic ischemic changes. The coronary angiogram showed ostial occlusion of the left anterior descending artery, which was associated with mid-right coronary artery embolic obstruction [24].

Limitations of studies examining the effects of high-dose AS on human health are notable. Pertinent information has often been published as case reports or short studies with insufficient control groups. Additionally, many studies did not specify the type and dosage of AS used. Furthermore, study participants may have been exposed to other performance-enhancing substances, which could distort the findings and heighten the risk of adverse effects. Moreover, it is anticipated that cardiac events associated with AS usage are underreported in medical literature due to individuals’ inclination to conceal their AS use, either for legal reasons in the case of athletes or to avoid social stigma. Consequently, the reported frequency of AS-related AMI may underestimate the true occurrence [17].

The pathogenic process of AMI caused by AS

To identify athletes at risk and prevent AMI and its repercussions, it is critical to understand the mechanisms underlying the occurrence of AMI in this population [16]. The potential mechanisms underlying AS-induced AMI can be categorized into atherogenic, thrombotic, and vasospastic factors.

Atherogenic Mechanism

Regarding the atherogenic mechanism, the abuse of AS can lead to a reduction in HDL-C and serum lipoprotein (a) levels, while increasing LDL-C levels [25, 26]. These changes, particularly significant reductions in HDL-C and HDL2-C, contribute to the atherogenic risk associated with AS, especially among long-term AS abusers. This is evident in reported cases of AS-related AMI in athletes, where AS abuse over several years was linked to underlying atherosclerosis. Notably, given that athletes engaged in aerobic exercise typically have higher HDL-C levels, a substantial decrease (22 mg/dl) raises suspicion of potential AS use [26]. Prolonged misuse of AS tends to elevate both systolic and diastolic blood pressure, although this is generally mild in magnitude [27]. Potential reasons for this rise may include the retention of sodium by the kidneys due to AS and the possibility of AS-induced vasospasm [28].

Thrombotic Mechanism

Experimental data in animals show that pretreatment with AS results in larger blood clots and shorter vessel-occlusion durations in response to thrombotic stimuli, suggesting that AS is thrombogenic. These effects are likely due to enhanced platelet aggregation, as AS have been found to amplify platelet aggregation through increased production of thromboxane A2, a potent platelet aggregator, and reduced production of prostacyclin, which inhibits platelet aggregation [17].

While AS consistently activates platelet aggregability, its impact on the coagulation cascade and fibrinolytic pathway is less clear [29]. Nevertheless, the observation of a high thrombus burden in the coronary arteries of AS users who have experienced AMI suggests that treatment with intravenous IIb/IIIa inhibitors is a reasonable option. Some case reports have also linked AS-related AMI to elevated homocysteine levels, which AS can increase, potentially promoting atherosclerosis and thrombosis in coronary arteries [17]. Additionally, AS can stimulate erythropoiesis, particularly in high doses, leading to an increase in hematocrit and blood viscosity, predisposing individuals to thrombosis. This is supported by a case report of AS-induced AMI with significant polycythemia (hemoglobin: 22 g/dl, hematocrit: 63%) [30]. Given the highly thrombogenic environment associated with AS use, it is advisable to thoroughly search for intracardiac thrombi, especially in AS users who experience AMI [17]. Thromboembolic events in the context of AS-induced AMI have been reported as strokes or renal infarctions.

Vasospastic Mechanism

The AS-induced coronary vasospasm is one logical explanation for the finding of patent coronary arteries in certain coronary angiograms and/or autopsy of AS users who experienced AMI [17]. In fact, long-term administration of AS to rabbits increased the aorta’s reactivity to vasoconstrictors and decreased its reaction to vasodilators. These effects were linked to a decrease in nitric oxide-mediated relaxation, which was caused by guanylate cyclase suppression [17]. Abuse of AS has also been shown to cause vascular dysfunction, impacting vasodilatation that is both endothelial-dependent and endothelial-independent [31]. From this vantage point, long-term AS therapy may exacerbate coronary vasospasm, even though research on AS’s impact on coronary vascular function is still lacking.

Cardiovascular complications of AS misuse

The cardiovascular system undergoes the impact of atherogenesis, increased blood clotting tendencies, and heightened oxygen requirements for the heart due to hypertrophy. In a review by Chang et al., the involvement of AS in coagulation, thrombus formation, and fibrinolysis was explored, revealing alterations in nearly all coagulation factor concentrations following steroid administration [32]. Multiple studies and case reports have presented instances of direct myocardial injury induced by AS, with autopsied hearts commonly showing left ventricular hypertrophy, often accompanied by fibrosis and myocytolysis [33]. Despite the likelihood of increased complications in AS users experiencing AMI, it is anticipated that AS-related cardiac events are underreported in medical literature due to socio-psychological factors, including the desire to conceal AS use for legal reasons and to avoid social stigmatization [23].

These substances have a profound impact on various crucial components of the cardiovascular system. Numerous studies have shown changes in lipid metabolism following the administration of AS, manifesting as an elevation in LDL cholesterol and a reduction in HDL cholesterol levels, ultimately leading to dyslipidemia - an integral factor in atherogenesis and cardiovascular diseases [26,34]. In terms of blood pressure and endothelial function, pivotal risk factors in cardiovascular diseases, the effects of AS remain a subject of debate. While certain animal model studies suggest that AS can reduce blood pressure by enhancing nitric oxide synthetase activity [35], several clinical studies indicate an increase in both systolic and diastolic blood pressure values following AS administration [28,34]. The rationale behind this phenomenon might be attributed to the neutralization of the nitric oxide effect at elevated doses, as reactive oxygen species (ROS) produced during heightened oxidative stress counteract its impact, leading to vasospasms coupled with substantial sodium retention [36]. The occurrence of hypercoagulability secondary to AS administration is a factual outcome, elucidated by the elevation in hemoglobin concentration, increased synthesis of thromboxane A2 and fibrinogen, and the inhibition of prostacyclin production [23,37].

AS Misuse and Mortality

The misuse of AS has a significant impact on mortality. In 2018, Helal et al. revealed that athletes using AS faced a risk of death 6-20 times higher than their non-AS-using counterparts [38]. Autopsy studies conducted by Frati et al. [39] (involving 19 cases) and Montisci et al. (involving four cases) [40], focusing on sudden deaths linked to AS misuse, uncovered a broad spectrum of macro- and microscopic alterations. These encompassed concentric hypertrophy, hypertrophic cardiomyopathy, myocarditis, focal fibrosis, peripheral venous thrombosis, pulmonary, renal, and hepatic thromboembolism, right ventricle dilatation, infarction with and without coronary occlusion, small-vessel disease, and intraventricular thrombosis. These findings underscore that, despite the recognized mortality risks associated with AS use, the full extent of the harms related to its misuse remains incompletely understood [4].

## Conclusions

Chest pain in a young patient population secondary to MI is not uncommon these days, and it is prudent to always evaluate drug history, including AS use. Athletes, bodybuilders, and others who use steroids or other drugs that are responsible for MI should be under the supervision of physicians so that the complications of steroids are ascertained, and if steroids are needed for any medical illness, proper dosage and follow-up should be emphasized. Therefore, while taking history from a patient, it is essential for physicians to be aware of the association between steroids and coronary artery disease.

In our patient, the temporal relationship between AS use and myocardial infarction, in the absence of atheromatous disease, strongly suggests a probable causal association. However, given additional contributing factors such as smoking and a positive family history, a multifactorial pathogenesis cannot be excluded. Physicians should always suspect AS abuse in a young adult suffering from MI, especially in the context of low HDL-C levels. It is worth noting that athletes rarely inform their physicians of AS use, and physicians rarely ask. This might be due to the limited knowledge of physicians regarding the recreational use of AS. Educational campaigns have become a pressing need to increase the public awareness of the serious complications of AS abuse. There is a dearth of scientific studies and documented cases of MI among young athletes in the literature that is currently available; as a result, more research on this subject is required.
